# A Lipid Nanoparticle-Based Method for the Generation of Liver-Specific Knockout Mice

**DOI:** 10.3390/ijms241814299

**Published:** 2023-09-19

**Authors:** Sumiyo Morita, Takuro Horii, Mika Kimura, Ryosuke Kobayashi, Hiroki Tanaka, Hidetaka Akita, Izuho Hatada

**Affiliations:** 1 Laboratory of Genome Science, Biosignal Genome Resource Center, Institute for Molecular and Cellular Regulation, Gunma University, 3-39-15 Showa-machi, Maebashi 371-8512, Japan; 2 Laboratory of DDS Design and Drug Disposition, Graduate School of Pharmaceutical Sciences, Tohoku University, 6-3 Aoba, Aramaki, Aoba-ku, Sendai 980-8578, Japan; 3 Viral Vector Core, Gunma University Initiative for Advanced Research (GIAR), 3-39-15 Showa-machi, Maebashi 371-8511, Japan

**Keywords:** knockout mice, Cre/loxP, lipid nanoparticles (LNPs)

## Abstract

Knockout mice are useful tools that can provide information about the normal function of genes, including their biochemical, developmental, and physiological roles. One problem associated with the generation of knockout mice is that the loss of some genes of interest produces a lethal phenotype. Therefore, the use of conditioned knockout mice, in which genes are disrupted in specific organs, is essential for the elucidation of disease pathogenesis and the verification of drug targets. In general, conditional knockout mice are produced using the Cre/loxP system; however, the production of the large numbers of Cre/flox knockout and control mice required for analysis requires substantial time and effort. Here, we describe the generation of liver-specific conditional knockout mice via the introduction of lipid nanoparticles encapsulating *Cre* mRNA into the liver of floxed mice. This technique does not require the production of offspring by mating floxed mice and is therefore more convenient than the conventional method. The results presented here demonstrate that the LNP-based method enables liver-specific gene knockout in a short period of time.

## 1. Introduction

**Figure 1:**
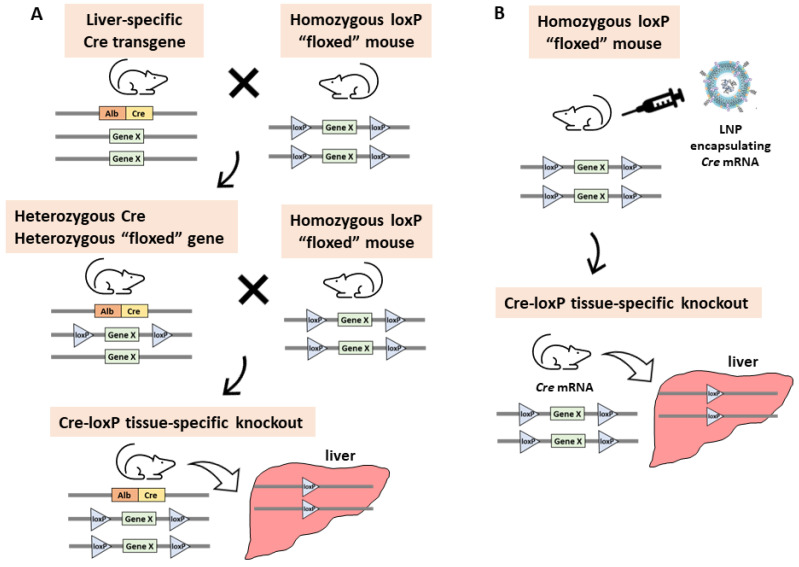
An overview of two strategies for the generation of liver-specific gene knockout mice. (**A**) The conventional method involving the two-stage crossing of *AlbCre* and floxed mice. (**B**) The newly developed method involving the use of LNP-encapsulated *Cre* mRNA.

The International Mouse Phenotyping Consortium (IMPC) is a global research infrastructure that generates and phenotypes knockout mice lines for protein-coding genes and determines the viability of homozygotes to assess gene essentiality [1,2,3]. According to the IMPC, approximately one-third of mouse gene knockouts are embryonic or perinatal lethal; hence, it is impossible to analyze the functions of these genes in adult mice. In addition, if genes are lost from the beginning of the developmental stages, the compensatory functions of other genes can make it difficult to understand the original function of the knockout gene. To overcome these problems, methods for the timing- or tissue-specific knockout of genes have been developed. The Cre/loxP system [4] is an approach for generating tissue-specific gene knockout mice. The standard method requires two different genetically engineered mouse strains; the first strain contains a targeted gene flanked by two loxP sites (floxed gene) in a direct orientation, and the second is a conventional transgenic mouse strain expressing *Cre* recombinase under the control of a tissue-specific promoter. To obtain tissue-specific knockout mouse models, two crosses of these strains are required (Figure 1). First, a *Cre* transgenic mouse and a flox mouse are crossed to generate *Cre*/*wt flox*/*wt* mice. Approximately 50% of the offspring will be heterozygous for the loxP allele and the *Cre* transgene. In the second step, a *Cre*/*wt flox*/*wt* mouse is crossed with a *flox*/*flox* or *flox*/*wt* mouse to obtain *Cre*/*wt flox*/*flox* conditional knockout mice. Approximately 25% or 12.5% of the progeny from this mating will be homozygous for the loxP allele and heterozygous for the *Cre* transgene. The overall procedure takes several months and requires much effort to generate the large number of mice required for analysis.

Lipid nanoparticles (LNPs) are a non-viral gene delivery system and were originally developed to deliver small-interfering RNA (siRNA) to the liver [5]. LNPs comprise phospholipids, sterols, polyethylene glycol (PEG)-conjugated lipids, and ionizable lipids. Since they were used in Pfizer-BioNTech and Moderna’s COVID-19 vaccines [6], LNPs are noted for their safety and effectiveness. LNPs can package large components such as mRNA [7], and they have been recently used for the introduction of the CRISPER/Cas system in vivo. When they are delivered intravenously, most of them are taken up by the liver [8]. To bypass the complicated and time-consuming mouse crossing procedure required to generate tissue-specific gene knockouts, we have developed an LNP-based method to deliver *Cre* mRNA to the liver of floxed mice. This method does not require the mating of floxed and *Cre* mice and was used successfully to generate liver-specific gene knockout mouse strains in less time than the conventional method.

## 2. Results

### 2.1. Generation of Liver-Specific-Tet3 and Meg3 Knockout Strains via Mice Crossing

We chose *Tet3* and *Meg3* as targets because we have been engaged in the study of imprinted genes and their regulation. As control experiments, we generated liver-specific knockout mice using the ordinary crossing method. *Tet3^flox/wt^* and *Meg3^flox/wt^* mice (C57/B6J background) were mated with *AlbCre* transgenic mice (C57/B6J background) in which *Cre* expression is controlled by a liver-specific albumin promoter. The *AlbCre^+/wt^ Tet3^flox/wt^* and *AlbCre^+/wt^ Meg3^flox/wt^* offspring were crossed with *Tet3^flox/flox^* and *Meg3^flox/flox^* mice to generate *AlbCre^+/wt^ Tet3^flox/flox^* and *AlbCre^+/wt^ Meg3^flox/flox^* mice, respectively. In these mice, *Cre* should be expressed in the liver only. To analyze Cre-mediated recombination, PCR analyses of the *Tet3* and *Meg3* alleles were performed in the liver, brain, lung, kidney, skeletal muscle, WAT, and testis samples (Table 1). Recombination-mediated deletion of the floxed allele occurred in the liver only, and the recombination rates were approximately 81% for *Tet3* and 63% for *Meg3*.

### 2.2. Generation of Liver-Specific Tet3 and Meg3 Knockout Strains via LNP-Mediated Delivery of Cre mRNA

*Tet3^flox/flox^* and *Meg3^flox/flox^* mice were dosed with LNPs encapsulating *Cre* mRNA (LNP-*Cre*) at 0.05 or 0.1 mg/kg body weight or dosed with DPBS as a negative control. Subsequently, Cre-mediated recombination was analyzed in liver, brain, lung, kidney, skeletal muscle, WAT, testis and spleen samples. As seen for the *AlbCre^+/wt^ Tet3^flox/flox^* and *AlbCre^+/wt^ Meg3^flox/flox^* mice, the recombination-mediated deletion of both floxed alleles occurred in the liver of LNP-*Cre*-treated mice (Table 2). The recombination rates of the *Tet3*- and *Meg3*-floxed alleles in mice dosed at 0.1 mg/kg body weight were approximately 79% and 54%, respectively. When the dose was reduced to 0.05 mg/kg, the recombination rates were decreased slightly to 73% and 44%, respectively. The LNP-*Cre*-mediated deletion was also detected in the lung, kidney, skeletal muscle, WAT, and spleen of *Tet3^flox/flox^* mice and *Meg3 ^flox/flox^* mice dosed at 0.05 or 0.1 mg/kg body weight (Table 2). As the recombination rate was at most 7% in mice dosed at 0.05 mg/kg body weight, liver-specificity was considered to be preserved to some extent.

### 2.3. Expression Analysis of Tet3 and Meg3 in the Liver

**Figure 2:**
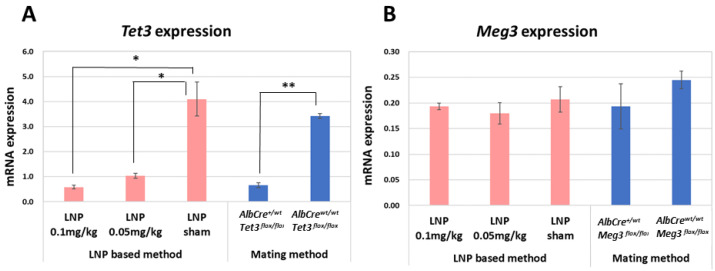
Relative expression levels of *Tet3* and *Meg3* in the liver of mice using the LNP-based method and mating method. (**A**) *Tet3* expression. (**B**) *Meg3* expression. Three mice were used in each group to measure expression in the liver, and qPCR analysis was performed in triplicate. The pink bars shows the gene expression using the LNP-based method and the blue bars shows the expression using the mating method. Significance was evaluated using Student’s *t*-tests. * *p* < 0.05, ** *p* < 0.01.

The expression levels of *Tet3* and *Meg3* in the liver of mice induced by the LNP-based method and mating method were determined by qPCR (Figure 2). Compared with that in LNP sham mice and *AlbCre^wr/wt^ Tet3^flox/flox^* mice, the expression level of *Tet3* was down-regulated by approximately 80% in the liver of both LNP-treated mice and *AlbCre^+/wt^ Tet3^flox/flox^* mice. The expression level of *Meg3* was down-regulated slightly (by approximately 10–20%) in the liver of LNP-treated and *AlbCre^+/wt^ Meg3^flox/flox^* mice.

### 2.4. Acute Liver Toxicity Evaluation

**Figure 3:**
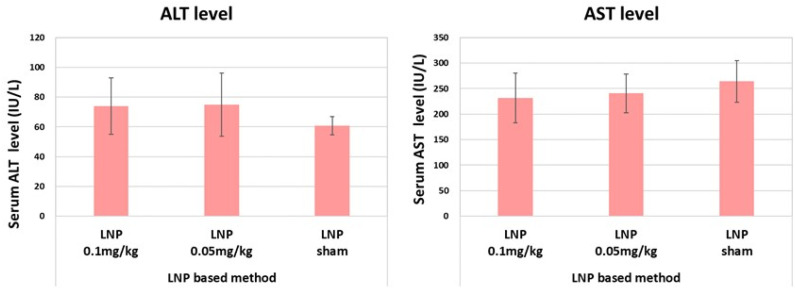
Hepatic toxicity of LNP. The ALT and AST levels were measured using C57B6/J mice (male 8 weeks), which were dosed with LNPs encapsulating *Cre* mRNA (LNP-Cre) at 0.05 or 0.1 mg/kg body weight or dosed with DPBS. Four mice were used in each group to measure serum ALT and AST levels. Significance was evaluated using Student’s *t*-tests.

Liver toxicity was measured using male C57B6/J (male 8 weeks old) mice dosed with LNPs encapsulating *Cre* mRNA (LNP-*Cre*) at 0.05 or 0.1 mg/kg body weight or dosed with DPBS (sham). There was no significant difference in liver enzyme (alanine transaminase, ALT and aspartate aminotransferase, AST) levels (Figure 3). 

## 3. Discussion

The Cre/loxP system is a reliable tool for generating tissue-specific knockout mice. However, the conventional technique requires at least two rounds of mating, and it takes several months to generate the large number of mice required for analysis. To overcome these difficulties, we developed an in vivo method for the introduction of LNP-encapsulated *Cre* mRNA into the liver of floxed mice to generate liver-specific conditional knockout mice, which does not require mating with *AlbCre* mice.

LNPs interact with serum proteins such as apolipoprotein E, facilitating their efficient uptake by hepatocytes in a receptor-mediated manner [9,10]. Here, we demonstrated that LNP-encapsulated *Cre* mRNA can be introduced efficiently into liver cells to generate liver-specific gene knockout mice in less time than the conventional method. The rates of deletion of the *Tet3*-floxed and *Meg3*-floxed alleles in the liver of LNP-*Cre*-treated mice were slightly lower than those in the *AlbCre^+/wt^ Tet3^flox/flox^* and *AlbCre^+/wt^ Meg3^flox/flox^* mice, respectively (Table 1 and Table 2). Nonetheless, *Tet3* expression was down-regulated to a similar extent in the liver of *AlbCre^+/wt^ Tet3^flox/flox^* mice and LNP-*Cre*-treated mice (Figure 2). *Meg3* expression was also down-regulated similarly, but only slightly, in the liver of *AlbCre^+/wt^ Meg3^flox/flox^* mice and LNP-*Cre*-treated mice. The recombination rate was only about 50% in the liver of these mice, which may explain the lack of a significant reduction in *Meg3* expression. Alternatively, gene expression of the non-recombinant alleles may have increased to compensate for changes in the gene expression of the recombinant alleles.

A recent study [11] investigated the most efficient LNP size for gene silencing in hepatocytes. The siRNA was designed to silence coagulation factor VII (siFVII), which is expressed in and secreted by hepatocytes. LNP-siRNA systems with different LNP diameters were produced (27 to 117 nm) and the silencing efficiency of FVII was analyzed. An LNP size of around 80 nm exhibited the most efficient gene silencing in the liver; therefore, we prepared LNPs with a size of around 80 nm.

In LNP–*Cre*-treated mice, deletion of the floxed allele occurred in tissues other than the liver; however, the recombination-mediated deletion rate in these non-target tissues was less than 7% when the mice were dosed at 0.05 mg/kg body weight. A previous study demonstrated that apolipoprotein E plays an important role in the hepatocellular uptake of LNP via the low-density lipoprotein receptor, and hepatocellular uptake was significantly decreased in apolipoprotein E knockout mice [6,7]. Thus, the binding of apolipoprotein E to the surface of LNPs followed by uptake via an LDL receptor is the major uptake mechanism for this particle. This receptor is expressed mainly in the liver, but it is also expressed in the lung, kidney, skeletal muscle, WAT, and testis, which explains why flox recombination occurred in these tissues.

Since mRNA does not have the ability to self-replicate, its expression is inevitably transient. Nevertheless, LNP–*Cre*-mediated recombination occurred to the same extent as in *AlbCre^+/wt^ Tet3^flox/flox^* and *AlbCre^+/wt^ Meg3^flox/flox^* mice after a single dose of LNP-*Cre*. Hence, we consider the method of producing liver-specific knockout mouse by administering LNPs to be very useful.

Successful adeno-associated vector (AAV)-mediated *Cre* expression has been reported in the liver [12,13], but it is associated with elevated serum levels of alanine aminotransferase and aspartate aminotransferase, indicating high liver toxicity compared with LNP administration [12,14]. AAV8 is suitable for liver-directed gene delivery [15]; however, to achieve liver-specific *Cre* expression, a hepatocyte-specific major urinary protein promoter must be used. The liver-specific knockout of genes has been achieved in vivo using CRISPR/Cas9 genome editing technology [16,17,18] alongside a viral delivery system such as AAV [19], or LNP [20]. Like the LNP-based method described here, use of the CRISPR/Cas9 system to generate liver-specific gene knockouts does not involve the crossbreeding of mice. Although the targeting specificity of Cas9 is believed to be controlled tightly by the 20 nt guide sequence of the sgRNA and the presence of a protospacer adjacent motif (PAM) adjacent to the target sequence in the genome, potential off-target cleavage can still occur on DNA sequences with even three to five base pair mismatches in the PAM–distal part of the sgRNA-guiding sequence. These off-target effects have to be considered in experiments using knockout mice generated using the CRISPR/Cas9 system. By contrast, there is no off-target effect in Cre-mediated loxP recombination.

Lipid nanoparticles (LNPs) have revolutionized the field of drug delivery through their ability to facilitate in siRNA delivery to the liver [5] and have been made popular through their use in the Pfizer-BioNTech and Moderna’s COVID-19 mRNA vaccines [6]. Since they can include long mRNA, they are ideal for mRNA-based gene therapy and the production of the RNA vaccine [21]. In addition, the advances in LNP formulation technology include the efficiency of inclusion and delivery of mRNA, allowing LNPs to be widely used for the in vivo delivery of exogenous mRNA, including the in vivo introduction of Cas9-mediated gene editors [22]. However, when injected intravenously, most of the LNPs are trapped by the liver. Modifying the lipid composition of the LNPs would allow more specific delivery to organs such as the brain, lung, muscle, heart, liver, spleen, and bone [23,24].

In conclusion, the results presented here demonstrate that the introduction of LNP-encapsulated *Cre* mRNA into floxed mice can be used to generate liver-specific conditional gene knockouts. Compared with the conventional method of mouse mating, this LNP-based method saves at least 6 months. Modifying the composition of the lipids in the LNPs would enable specific delivery to various organs and the generation of various tissue-specific knockout mice.

## 4. Materials and Methods

### 4.1. Animals 

**Figure 4:**
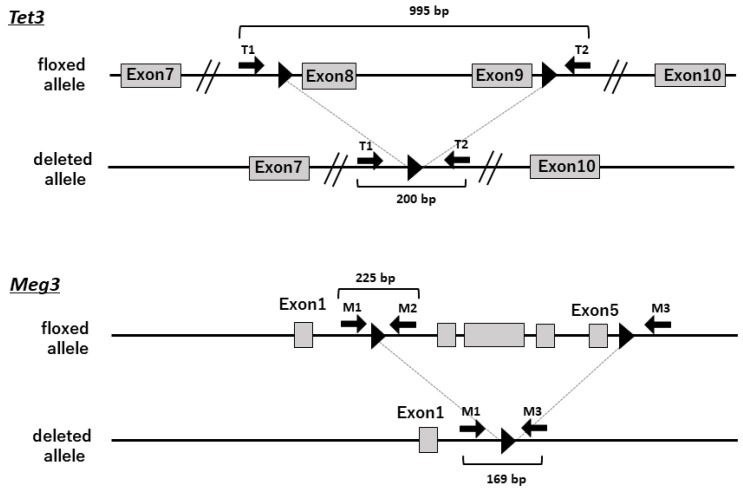
Gene-targeting strategy. Exons of the murine *Tet3* and *Meg3* genes are represented by square boxes, and the loxP site is indicated by a black arrowhead. The floxed allele and deleted allele are shown. Primers used for genotyping of the floxed and deleted alleles are shown as arrows.

Male C57BL/6J mice aged 8 weeks were purchased from Charles River Japan (Yokohama, Japan). *Tet3*-flox and *Meg3*-flox mice were generated using the CRISPR/Cas9 system (Figure 4) [25]. Briefly, to create *Tet3*-floxed mice, two gRNAs targeting *Tet3* intron 7 and intron 9 were designed as well as corresponding lox site ssODNs with 60 bp homology to sequences on each side of each gRNA-mediated double strand break (Supplementary Table S1). To create *Meg3*-floxed mice, two gRNAs targeting *Meg3* intron 1 and intron 5 and two loxP site ssODNs with 60 bp homology sequences were also designed (Supplementary Table S1). Each gRNA and each ssODN was sequentially introduced by electroporation with Cas9 recombinant protein at 1-cell and 2-cell embryonic stages, respectively, as described in [25]. *AlbCre* mice [26] were obtained from the Jackson Laboratory (Bar Harbor, ME).

### 4.2. PCR Analyses of the TET3, Meg3, and AlbCre Alleles

Genomic DNA was extracted from the mouse tail using the conventional phenol/chloroform method. To detect the *Tet3*-floxed allele, PCR was performed using the following primers (Supplementary Table S1): left loxP: Tet3 Ex8-1 and Tet3 Ex8-2; right loxP: Tet3-F and Tet3-R. To detect the *Meg3*-floxed allele, PCR was performed with the following primers: left loxP: Meg3-L1 and Meg3-L2; right loxP: Meg3-R3 and Meg3-R4. To detect the *AlbCre* allele, PCR was performed with the primers 20239, 20240, and IMR5374.

### 4.3. PCR Analyses of Floxed and Deleted Alleles in Mouse Tissues

Genomic DNA was prepared from the liver, brain, lung, kidney, skeletal muscle, white adipose tissue (WAT), and testis of mice aged 8–10 weeks. Three animals were used for each of the *AlbCre^+/wt^ Tet3^flox/flox^* mice and their corresponding control *AlbCre^wt/wt^ Tet3^flox/flox^* mice as well as for *AlbCre^+/wt^ Meg3^flox/flox^* mice and their corresponding control *AlbCre^wt/wt^ Meg3^flox/flox^* mice.

To detect the *Tet3*-floxed allele (995 bp) and deleted allele (200 bp), PCR was performed with T1 and T2 primers (Figure 4, Supplementary Table S1). To detect the *Meg3*-floxed allele (225 bp) and deleted allele (169 bp), PCR was performed with primers (Figure 4, Supplementary Table S1) designed to amplify the *Meg3*-floxed alleles M1 and M2 and deleted alleles M1 and M3. The amplified DNA was electrophoresed, and the densities of the bands corresponding to each allele were quantified by densitometry.

Because the PCR amplification efficiencies of the floxed allele (long) and deleted allele (short) were different, we determined the amplification efficiency of each allele by measuring the intensities of the PCR fragments generated with an equimolar mixture of each allele DNA in advance (Supplementary Materials and Methods). Recombination efficiency was determined based on the PCR fragment intensities normalized by the amplification efficiency of the floxed allele and the deleted allele (Supplementary Tables S2 and S3).

### 4.4. Quantitative RT-PCR Analyses

Total RNA was isolated from the liver of mice aged 8–10 weeks using TRIzol reagent (Invitrogen, Carlsbad, CA, USA). Gene expression levels were measured using a LightCycler 96 system (Roche, Basel, Switzerland) and SYBR Premix Ex Taq (Takara Bio, Kusatsu, Japan), according to the manufacturers’ instructions. Expression levels were normalized against those of 18S ribosomal RNA. PCR was performed with the following primers (Supplementary Table S1): *Tet3*: Tet3-1, Tet3-2; *Meg3*: Meg3-1, Meg3-2; *18S*:18s-F, 18s-R. Three mice were used per group to measure expression in the liver, and qPCR analysis was performed in triplicate.

### 4.5. Preparation of LNPs

The mRNA encoding *Cre* (5 mo modified, cat no L-7211-100; Trilink BioTechnologies, San Diego, CA, USA) was diluted at a concentration of 0.0067 μg /μL in 20 mM malic acid buffer (pH 3.0, with 30 mM NaCl). The lipid ethanol solution was prepared at a concentration of 4 mM. The lipid component was COATSOME^®^SS-OP/DOPC/cholesterol (54.6/7.8/4.2) with an additional 1.5 mol% of DMG-PEG2000. COATSOME^®^SS-OP, DOPC, and DMG-PEG2000 were obtained from NOF Corporation (Kawasaki, Japan). These solutions were mixed using the NanoAssemblr Ignite TM system (total flow rate: 4.0 mL/min; flow rate ratio: water/ethanol = 3/1 (*v*/*v*)) [11]. The mixture was recovered and mixed with an equal volume of 20 mM MES buffer (pH 6.0). The external solution was replaced with DPBS by ultrafiltration using Amicon Ultra-15 (100 K) (Merck, KGaA, Germany) centrifugal units. The size, polydispersity index (PdI), and zeta potential of LNPs were measured by dynamic light scattering (Zetasizer nano ZS; Malvern Panalytical, Worcestershire, UK). The encapsulation ratio of mRNA was measured using the Qubit RNA HS Assay kit. The properties of the in vivo dosed LNP are shown in Table 3 and Supplementary Figure S1. 

Mice were dosed with LNPs encapsulating *Cre* mRNA (LNP-*Cre*) at 0.05 or 0.1 mg/kg body weight via the jugular vein. Sham control mice were dosed with DPBS (0.2 mL per mouse). In each group, three mice were used for analysis. 

### 4.6. Liver Toxicity Measurement

Liver toxicity was measured using C57B6/J mice (male 8 weeks), which were dosed with LNPs encapsulating *Cre* mRNA (LNP-*Cre*) at 0.05 or 0.1 mg/kg body weight or dosed with DPBS. At 24 h after administration, blood samples were collected from the jugular vein. The ALT and AST levels were measured with L type WAKO ALT J2 and L type WAKO AST-J2 (FUJIFILM Wako Pure Chemical, Osaka, Japan) according to the manufacturer’s instructions.

### 4.7. Statistical Methods

Recombination efficiency of the floxed allele and mRNA expression were analyzed by Student’s *t*-test (two-tailed) for pairwise comparisons. Data are presented as mean and S.D. *p* < 0.05 was considered significant. 

## Figures and Tables

**Table 1:** The rates (%) of deletion of the *Tet3*-floxed and *Meg3*-floxed alleles in tissues of *AlbCre^+/wt^ Tet3^flox/flox^* mice and their corresponding control *AlbCre^wt+/wt^ Tet3^flox/flox^* mice, and in tissues of *AlbCre^+/wt^ Meg3^flox/flox^* mice and their corresponding control *AlbCre^wt/wt^ Meg3^flox/flox^* mice. WAT; white adipose tissue. n.d.; not detectable. Data are presented as the mean ± S.D. (*n* = 3).

	The Rate (%) of Deletion of Floxed Allele						
	Liver	Brain	Lung	Kidney	Sk. Muscle	WAT	Testis
*AlbCre^+/wt^ Tet3 ^flox/flox^*	81.4 ± 2.1	n.d.	n.d.	n.d.	n.d.	n.d.	n.d.
*AlbCre^wt/wt^ Tet3 ^flox/flox^*	n.d.	n.d.	n.d.	n.d.	n.d.	n.d.	n.d.
*AlbCre^+/wt^ Meg3 ^flox/flox^*	62.8 ± 1.3	n.d.	n.d.	n.d.	n.d.	n.d.	n.d.
*AlbCre^wt/wt^ Meg3 ^flox/flox^*	n.d.	n.d.	n.d.	n.d.	n.d.	n.d.	n.d.

**Table 2:** The rates (%) of deletion of the *Tet3*-floxed and *Meg3*-floxed alleles in tissues of *Tet3^flox/flox^* and *Meg3^flox/flox^* mice administrated LNPs encapsulating *Cre* mRNA. WAT; white adipose tissue. n.d.; not detectable. Data are presented as the mean ± S.D. (*n* = 3).

	The rate (%) of Deletion of Floxed Allele								
	Dose (mg/kg)	Liver	Brain	Lung	Kidney	Sk. Muscle	WAT	Testis	Spleen
*Tet3 ^flox/flox^*	0.1	78.8 ± 1.2	n.d.	n.d.	n.d.	n.d.	1.7 ± 1.6	n.d.	19.2 ± 2.4
	0.05	73.3 ± 2.1	n.d.	n.d.	n.d.	1.4 ± 1.4	1.6 ± 0.9	n.d.	7.0 ± 0.4
	sham	n.d.	n.d.	n.d.	n.d.	n.d.	n.d.	n.d.	n.d.
*Meg3 ^flox/flox^*	0.1	54.3 ± 2.4	n.d.	0.3 ± 0.3	0.8 ± 0.4	n.d.	1.4 ± 0.1	n.d.	5.3 ± 0.3
	0.05	44.0 ± 2.3	n.d.	n.d.	n.d.	n.d.	n.d.	n.d.	1.7 ± 0.2
	sham	n.d.	n.d.	n.d.	n.d.	n.d.	n.d.	n.d.	n.d.

**Table 3:** Properties of the LNP used in the in vivo experiments. Data are presented as the mean ± standard deviation (S.D.) (*n* = 3). Each LNP distribution was measured three times, and the data are shown in Figure S1.

Size (d, nm)	PdI	Zeta Potential (mV)	Recovered mRNA (%)
79.02 ± 1.40	0.1 ± 0.03	−1.27 ± 0.22	88.35 ± 5.59

## Data Availability

Not applicable.

## Supplementary Materials

The following supporting information can be downloaded at: https://www.mdpi.com/article/10.3390/ijms241814299/s1.
