# Supplementary material for: A Lipid Nanoparticle-Based Method for the Generation of Liver-Specific Knockout Mice
Source: Int J Mol Sci. 2023 Sep 19;24(18):14299. doi: 10.3390/ijms241814299 (PMC10532048; doi:10.3390/ijms241814299)
Supplement: Supplementary file 1 [file ijms-24-14299-s001.zip › Figure S1_SumiyoMorita_v2.pptx]

## Slide 1
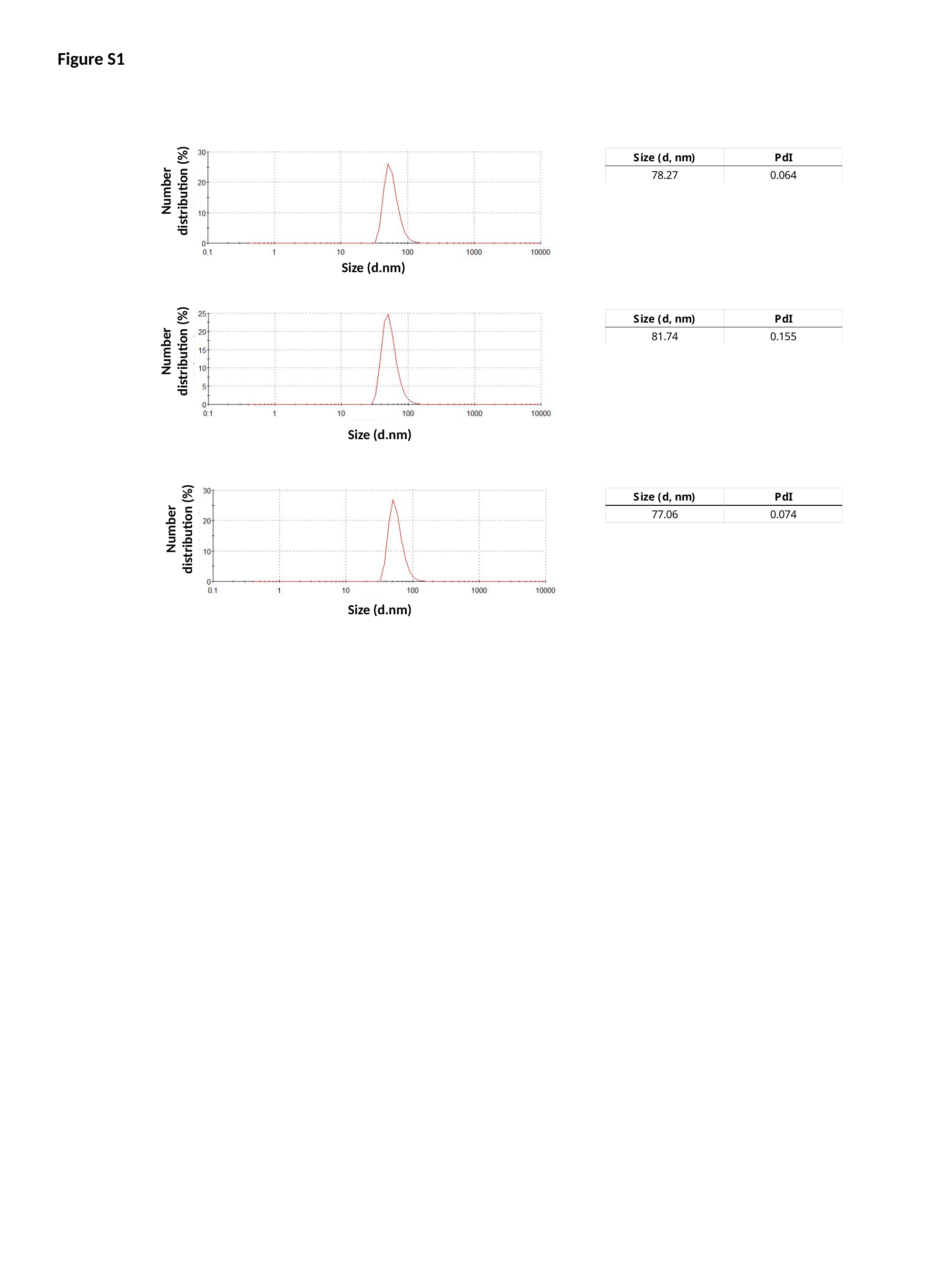

Figure S1
Number distribution (%)
Size (d.nm)
Number distribution (%)
Size (d.nm)
Number distribution (%)
Size (d.nm)

## Slide 2
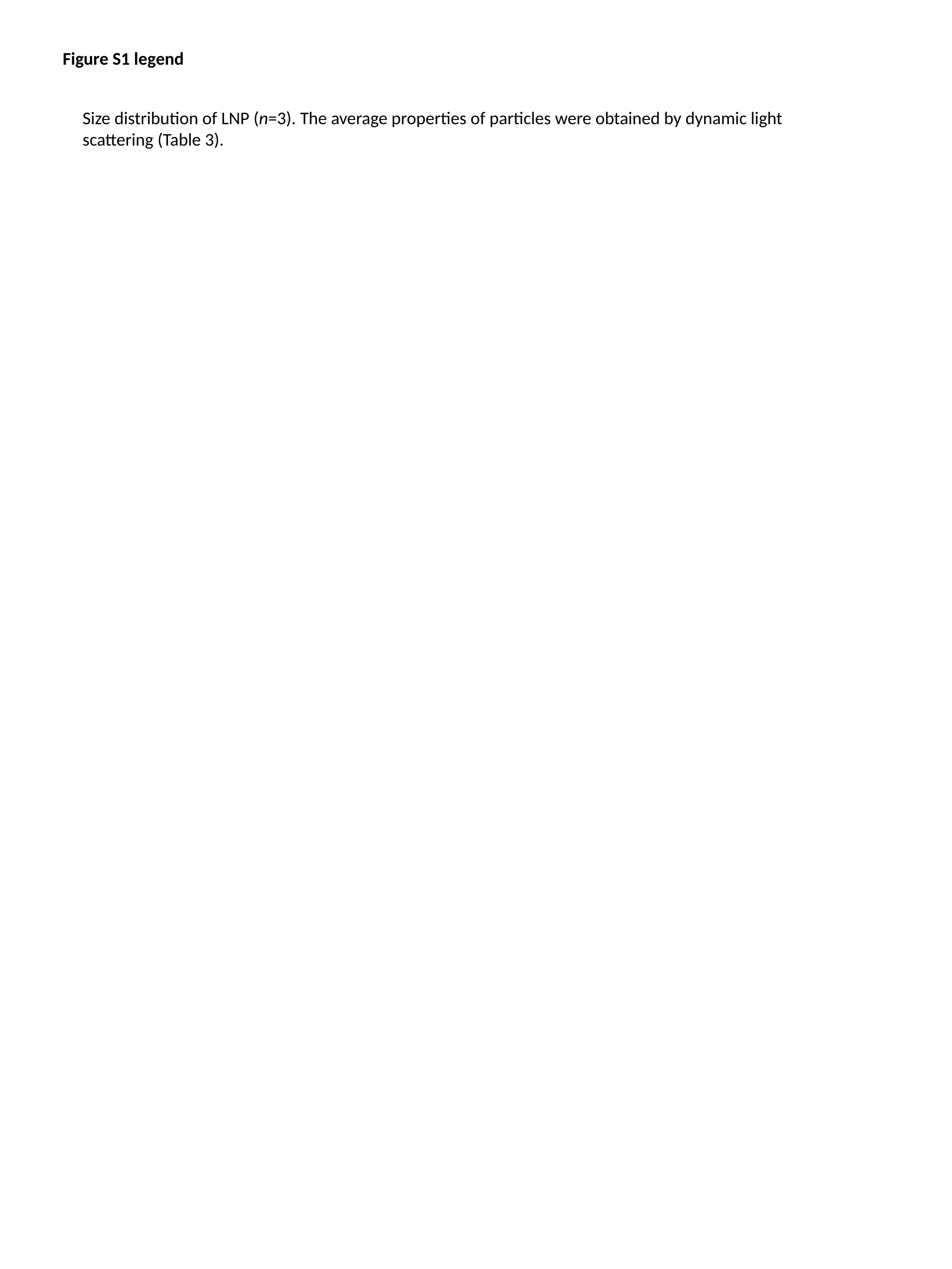

Figure S1 legend
Size distribution of LNP (n=3). The average properties of particles were obtained by dynamic light scattering (Table 3).
