# Supplementary material for: A Lipid Nanoparticle-Based Method for the Generation of Liver-Specific Knockout Mice
Source: Int J Mol Sci. 2023 Sep 19;24(18):14299. doi: 10.3390/ijms241814299 (PMC10532048; doi:10.3390/ijms241814299)
Supplement: Supplementary file 1 [file ijms-24-14299-s001.zip › Supplementary Materials and Method_SumiyoMorita_230910.pdf]

Supplementary Materials and Methods

The determination of PCR amplification efficiencies of the floxed allele and deleted allele.

1. Tet3

The *Tet3*-floxed allele (995 bp) and deleted allele (200 bp) were PCR-amplified with T1 and T2 primers (Figure 4, Supplementary Table S1). The amplification efficiency of each allele was determined by measuring the intensities of the PCR fragments amplified from an equimolar mixture of each cloned allele DNA. The PCR conditions are listed in Supplementary Table S1. The amplified DNA was electrophoresed on an agarose gel and the densities of the bands corresponding to each allele were quantified with Image J. PCR analysis was performed in triplicate as follows.

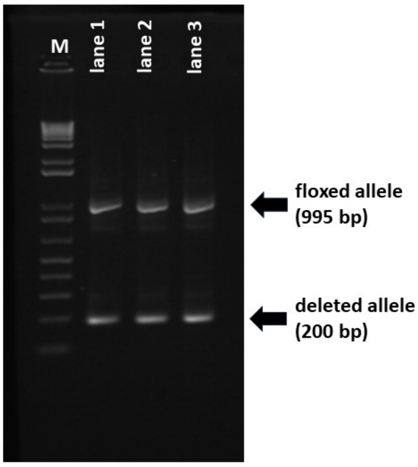

M: 1kb plus size marker

	Allele	Size (bp)	Densities of the bands	C(del/flox)	Average C
lane1	floxed	995	4496.861	1.0	1.0
	deleted	200	4460.719		
lane2	floxed	995	4576.619	1.0	
	deleted	200	4793.841		
lane3	floxed	995	4959.861	1.0	
	deleted	200	5137.962		

The C value is defined as the density of the deleted allele divided by the density of the floxed allele. This means that the deleted allele is amplified C times as much as the floxed allele; therefore, in subsequent analysis, we corrected the density of the floxed allele by multiplying C. Finally, recombination efficiency was calculated as follows; recombination efficiency (%) = (deleted allele x 100) / (deleted allele + floxed allele x C).

2. Meg3

The *Meg3*-floxed allele (225 bp) and deleted allele (169 bp) were PCR-amplified with mixed primers (Figure 4, Supplementary Table S1) designed to amplify the *Meg3*-floxed alleles (M1 and M2) and the deleted alleles (M1 and M3). We determined the amplification efficiency of

each allele by measuring the intensities of the PCR fragments amplified from an equimolar mixture of each allele DNA. The PCR conditions are listed in Supplementary Table S1. The amplified DNA was electrophoresed with MultiNA (Shimadzu, Kyoto, Japan) which is a microchip electrophoresis system, and the densities of the bands corresponding to each allele were quantified. PCR analysis was performed triplicate as follows.

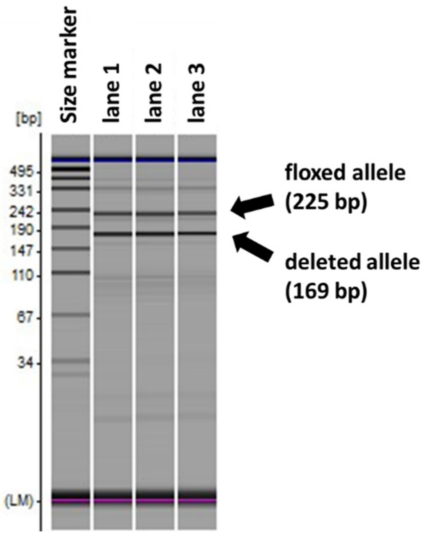

	Allele	Size (bp)	Densities of the bands	C(del/flox)	Average C
lane1	floxed	225	210.19	1.9	1.9
	deleted	169	390.66		
lane2	floxed	225	276.33	1.8	
	deleted	169	509.29		
lane3	floxed	225	214.57	1.9	
	deleted	169	397.34		

The C value and recombination efficiency were calculated in the same way as for Tet3.
